# Relationship Between Laterality of Orthodontic Miniscrew Failure and Clinical Variables

**DOI:** 10.3390/jcm13237115

**Published:** 2024-11-25

**Authors:** Aiko Ishizaki-Terauchi, Yuji Ishida, Makiko Okuzawa-Iwasaki, Chiyo Shimizu-Tomoda, Takashi Ono

**Affiliations:** 1 Department of Orthodontic Science, Graduate School of Medical and Dental Sciences, Institute of Science Tokyo (Science Tokyo), Tokyo 113-8549, Japan; ishizakiaiko0515@gmail.com (A.I.-T.); makiko101ok@gmail.com (M.O.-I.); chiyo85.orts@gmail.com (C.S.-T.); 2 Private Practice, Tokyo 156-0053, Japan

**Keywords:** laterality, miniscrew, motorized, operator, orthodontic treatment, dominant hand

## Abstract

**Background/Objectives**: Many reports on orthodontic miniscrew insertion focus on insertion conditions, such as technique, but not on the insertion environment, such as the operator’s dominant hand. This study aimed to ascertain whether the operator’s dominant hand affects laterality in the success rate of manual and motorized miniscrew insertion methods. **Methods**: This study retrospectively investigated the medical records of 250 Japanese patients, aged ≥15 years, who underwent miniscrew implantation in the maxilla by right-handed operators with at least 3 years of experience. A cross-tabulation analysis, Chi-square test, and multiple logistic regression analysis were performed to compare the success rates of the two insertion methods. **Results**: A total of 454 screws were implanted (346 manual and 108 motorized), with an overall success rate of 79.2%. No significant difference was observed in the success rate between the manual (77.7%) and motorized groups (84.2%). Regarding the laterality of the overall success rate, the right-side success rate (84.1%) was significantly higher than that of the left side (75.6%). The right-side success rate in the manual group (82.7%) was significantly higher than that of the left-side (73.0%). In the motorized group, no significant difference was observed between the success rate of insertion on the right (84.6%) and left sides (83.9%). Multiple regression analysis showed that the miniscrew insertion stability was influenced by the insertion side and the operator’s years of experience. **Conclusions**: The manual screw insertion method was susceptible to environmental factors and less successful in left-side insertion by right-handed operators.

## 1. Introduction

In orthodontic treatment, anchorage management is an important aspect [1] of treatment planning and progress, especially in cases involving tooth extraction. For several decades, anchorage control has usually been achieved using extraoral anchorage devices, such as headgear, and intramaxillary anchorage devices, including lingual, holding, and transpalatal arches. However, patient cooperation is essential to achieve the expected treatment objective; therefore, orthodontists have always struggled to manage anchorage control [2]. Since the 1980s, orthodontic skeletal anchorage devices have been developed [3], and orthodontic miniplates and miniscrews have been used as efficient temporary anchorage units without the need for patient cooperation. Miniscrews are now widely used in orthodontic treatment because they are less invasive than plates during insertion and removal and cause less discomfort during use [4,5].

However, complications associated with miniscrew placement, such as dislodgement and loss, have also been reported [6,7,8]. Risk factors for miniscrew dropout include age, sex, vertical facial morphology, oral hygiene, cortical bone thickness, quality of the alveolar bone, miniscrew length, insertion torque and angle, placement position, and contact with roots and vessels [6,7,8]. According to previous studies, the general success rate was estimated to be approximately 70–80%, and Kim et al. reported a significantly higher success rate with motorized screwdrivers than with manual screwdrivers [9].

Various environmental factors also influence medical procedures. Previous studies have reported that left–right differences exist in the treatment efficiency and results of dental procedures, depending on the operator’s dominant hand. Baqain et al. [10] reported a four-fold increase in the incidence of alveolar osteitis after extraction of the third molar on the side contralateral to the operator’s dominant hand, as well as a longer treatment time. Petsos et al. [11] also reported that the probing pocket depth and periodontal condition of the adjacent second molar after third-molar extraction was affected on the side contralateral to the operator’s dominant hand, indicating that the outcomes of dental procedures might be influenced by the operator’s dominant hand. In orthopedic surgeries such as hip arthroplasty, left–right differences in joint position and fit occur depending on the surgeon’s dominant hand, and some have reported that the use of a robot reduces the left–right differences [12,13]. However, this remains controversial.

Many reports on orthodontic miniscrew insertion focus on insertion conditions, such as technique. There are, however, no studies in the literature that focus on the insertion environment, such as the operator’s dominant hand, or the placement environment—including the shape and layout of the dental chair—and its position relative to the operator. In this study, we hypothesized a correlation between failure of orthodontic mini-screw insertion and the operator’s dominant hand, suggesting potential laterality. Therefore, we investigated the success rate of screw insertion by right-handed operators on both the left and right sides of a dental chair, comparing the laterality in the success rates between manual and motorized miniscrew placements.

## 2. Materials and Methods

### 2.1. Data Collection

This retrospective study was approved by the appropriate institutional Ethics Review Committee (approval number: D2022-054) and was conducted in accordance with the Declaration of Helsinki.

Orthodontic miniscrews were placed in 250 Japanese patients (53 males and 197 females; age range, 15–51 years; mean age: 26.6 years) who visited the orthodontic clinic between April 2017 and December 2020, by right-handed experienced operators with more than 3 years of experience. The patients were divided into two groups according to the miniscrew insertion method (a total of 454 miniscrews were placed): manual (346) and motorized (108). Cases were included in the study if orthodontic miniscrews were placed on the buccal side of the alveolar bone between the maxillary second premolar and the first molar or between the first and second molars. Of these patients, 51 were in their teens, 139 were in their 20s, and 65 were 30 years or older (Table 1). The exclusion criteria were as follows: diseases affecting bone metabolism (e.g., osteoporosis and diabetes mellitus), medications affecting bone metabolism, and moderate or severe periodontal disease (periodontal pockets > 4 mm).

**Figure 1:**
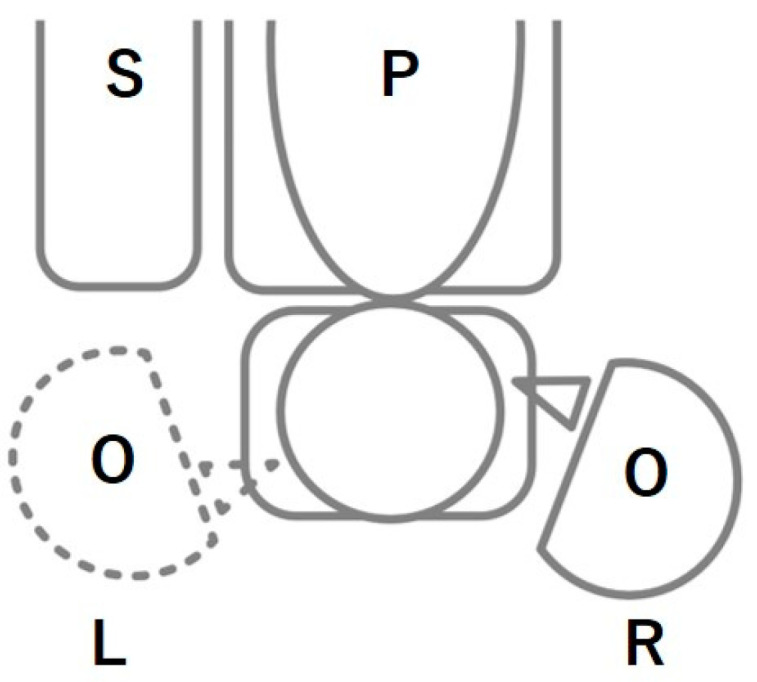
An illustrative schema of the position between the patient and the operator during miniscrew insertion. For insertions on the patient’s right side, the right-handed operator was positioned on the R (indicated by the solid semicircle). Conversely, for insertions on the left side, the operator stood on the L (marked by the dotted semicircle). Abbreviations: S, spittoon; P, patient; O, operator; R, right; L, left.

The miniscrews used were of the Dual-Top Anchor Screw auto screw III JA type (Jeil Medical Co., Seoul, Korea), 6 and 8 mm in length and 1.4 and 1.6 mm in diameter. A manual screwdriver (Jeil Medical Co., Seoul, Korea) or a battery-rechargeable motorized torque driver (Orthonia, 111-ED-010, Jeil Medical Co.) was used for placement. The miniscrews were placed at a rotational speed of 8–16 rpm and a torque of 10–15 Ncm with the motorized screwdrivers. All miniscrews were self-drilled, without raising a flap, under local infiltration anesthesia. After insertion, patients were administered analgesics, antibiotics, and 0.12% chlorhexidine mouthwash (Figure 1) [9,14].

### 2.2. Statistical Analyses

Instances of spontaneous dislodgement within six months after the insertion and cases where removal was necessary due to screw instability, movement, or pain were categorized as failures. A cross-tabulation analysis was performed for variables such as insertion method (manual or motorized), sex, age, and insertion site (between second premolars and first molars or between first and second molars), and the respective success rates were compared using the chi-square test. For the insertion sites, each intermolar site was examined separately. Multiple logistic regression analyses were performed to estimate the influence of each clinical variable on the success rate. Age, sex, insertion side, insertion site, and the years of experience of the operator were independent variables, and miniscrew insertion success was the dependent variable. The values of the independent factors are listed in Table 2. The odds ratio (OR) of each factor for miniscrew insertion success was calculated. Statistical significance was set at *p* < 0.05. All statistical analyses were performed using GraphPad Prism ver. 8.02 (GraphPad Software, San Diego, CA, USA).

## 3. Results

The overall success rate was 79.2%, with 454 miniscrews implanted (manual insertion, 346 and motorized insertion, 108). No significant difference was observed in the success rate between the manual (269/346; 77.7%) and motorized groups (91/108; 84.2%) (Table 3).

Regarding sex, the success rates for the males (59/74, 79.7%) and females (210/272, 77.2%) in the manual group showed no significant differences. In the motorized group, as in the manual group, there were no significant differences in the success rates between females (68/80, 85.0%) and males (23/28, 82.1%) (Table 4).

For patients younger than 20 years, the overall success rate was 79.1%, with no significant difference between the manual (60/76, 78.9%) and motorized groups (12/15, 80%) (Table 5). In patients aged 20–30 years, the overall success rate was 79.2%, with no significant difference between the manual (136/176, 77.2%) and motorized groups (62/74, 83.7%). Among those over 30 years of age, the overall success rate was 79.6%, with no significant difference between the manual (73/90, 81.1%) and motorized groups (17/19, 89.4%). No significant differences were observed in the success rates among the three age groups. Additionally, no significant difference in success rates was found between the two methods in all age groups.

Regarding the insertion side, the overall success rate on the right side (185/220, 84.1%) was significantly higher (*p* = 0.049) than that on the left side (177/234, 75.6%) (Table 6). In the manual group, the success rate on the right side (139/168, 82.7%) was significantly (*p* = 0.038) higher than that on the left side (130/178, 73.0%). However, no significant difference was observed in the success rates between the right (44/52; 84.6%) and left (47/56; 83.9%) sides in the motorized group.

Regarding the site of insertion, between the second premolar and first molar in the manual group, the success rate on the left side (113/157, 71.9%) was significantly lower (*p* = 0.03) than that on the right-side (127/153, 82.3%) (Table 7). Furthermore, in the manual group, regarding the first and second molars, there was no significant difference in the success rates for the left (17/21, 80.9%) and right (13/15, 86.6%) sides. However, there was no significant difference in the success rates on the left and right sides for the second premolar and first molar or the first and second molars in the motorized groups.

Multivariate analysis showed that the insertion side (OR, 0.602; 95% confidence interval [CI], 0.3754–0.9573) and the operator’s years of experience (OR, 2.875; 95% CI, 1.202–8.534) were related to the success rate of miniscrew insertion (Table 8). In this study, third- and fourth-year surgeons were compared with senior surgeons.

## 4. Discussion

We performed a retrospective study that evaluated the relationship between the laterality of orthodontic miniscrew failure and clinical variables. We observed no difference in the success rates of miniscrew insertion based on sex, which is consistent with the results of a previous study [15]. Chen et al. [16] noted an elevated failure risk in younger patients; however, we observed no age-related differences, irrespective of the insertion method. This could be because the sample was not adjusted for age during the sampling process. Previous studies have reported the success rate of anchor screw placement to be as high as 86.8%; however, we observed a success rate of 77.7% in the manual group and 84.2% in the motorized group. Therefore, the motorized group in this study had the same level of success as that reported in the literature, whereas the manual group tended to have lower success. A previous report suggested a positive correlation between patient age and screw success rate [17]. In this study, case–control comparison for the age of each group was not conducted; there were 76 teenagers in the manual group (21.9%) and 15 in the motorized group (13.8%), and it could be inferred that the manual group, with a higher percentage of teenagers, had a lower success rate. Furthermore, a previous study showed that the success rate was higher for Caucasians compared to Asians [18]. The lower success rate in this study may, therefore, have been influenced by the fact that the sample was wholly Japanese.

Regarding the insertion technique, the manual screwdriver insertion method exhibited instability in screwing speed, torque, and direction during placement. Furthermore, excessive mechanical pressure or unstable force may damage the bone and periodontal tissues at the insertion site [19]. Previous studies have reported that excessive torque application during screw insertion can cause microcracks in the alveolar bone around the screw or screw threading [20,21]. According to a report, the proximity of the screw to the tooth root affects its stability [8]. The results of our study were similar to those of this report, suggesting that the left side, which was not the dominant hand in the manual insertion method, may have had an unstable insertion direction and excessive pressure compared to the motorized group, as well as a higher likelihood of proximity to the tooth root, resulting in a higher failure rate and probability of trauma [22].

In this study, laterality in the success rate of screw placement by right-handed operators was investigated. Kim et al. [9] observed a significantly higher success rate (85.4%) in a motorized group compared to that in a manual group (74.8%). In this study, the success rate on the left side with the manual driver was 73.0%, which is similar to that reported in previous studies [9,23,24], whereas the success rate on the right side with the manual driver was 82.7%, which is comparable to the success rate of motorized drivers in this study (right side: 84.6%, left side: 83.9%) and previous studies on motorized drivers. We observed no difference in the success rate between each side in the motorized group and the right side in the manual group, which differs from the report by Kim et al. [9], suggesting that some laterality exists in the success rate of screw placement with manual drilling by hand dominance. However, hand dominance may not influence the success rate when performing motorized insertion. Right-handed operators often turn to the right relative to the dental chair when using a manual screwdriver to place a screw on the right side and secure the dominant arm by keeping the arm close to the body. However, during left-side insertion, maintaining arm proximity to the body and stabilizing posture, as well as handling the dominant arm, can be challenging due to the relative positioning difficulty with the dental chair. Previous studies have reported that, during mandibular third-molar extraction by right-handed operators, the extraction time on the left side is prolonged, and the periodontal condition after extraction is affected [10,11]. In orthopedic surgery, it has also been reported that left–right differences occur depending on the dominant hand of the surgeon [12,13]. In the present study, multiple logistic regression analysis also suggested that the insertion side was a factor affecting the success rate. The space on the left side of the chair is often narrower than that on the right side, likely resulting in an unstable position of the operator during insertion procedures. In the motorized method, the practitioner is usually positioned at the 12 o’clock position of the dental chair, regardless of left- or right-side insertion. Motorized insertion can help maintain a constant screw speed, torque, and direction during insertion. In this study, the success rate within the manual group may be influenced by the insertion side, whereas this effect was not observed in the motorized group.

Although many studies have compared the success rate of screw insertion between the maxillary and mandibular alveolus, buccal and lingual alveolus, and midpalate [14,25], few reports exist on interradicular site-specific success rates. In this study, when the success rates of each interdental site were examined separately, there were no significant differences in the success rates of screw placement in the left and right intermolar sites of the first and second molars in the motorized and manual groups. This may be due to the small sample size.

Hence, stable placement can be achieved using a motorized screwdriver, especially with screw insertion on the opposite side of the dominant hand. Further, Baqain et al. [10] reported that the non-dominant hand side has a poorer field of vision. Considering the potential impact of dominant hand positioning, the unit design should accommodate operator positioning and posture (e.g., a pediatric dental chair unit allowing unrestricted chairside movement) to mitigate environmental influences.

This study had some limitations. First, the investigation focused on procedures performed only by right-handed operators because the number of left-handed clinicians was insufficient to validate their success rate. For a more accurate investigation, differences in success rates between manual and motorized insertions performed by left-handed surgeons should also be examined. Second, because of the limited number of insertions between the first and second molars in this study, the test for the interradicular site-specific effect on the success rate lacked strong validity. Finally, a case–control comparison of patients’ ages between the two experimental groups was not possible. The influence of the age components of each group cannot be eliminated from the current results, especially in the investigation of the differences in insertion methods. In this study, the surgeon’s implantation position was changed depending on the side of implantation, but rotating the patient’s head to the right without changing the surgeon’s implantation position during left-sided implantation may affect the left-sided success rate. It is also possible that the type of unit used may have affected the results of this study. In addition, Hoi-Jeong et al. [26] reported a higher success rate for those with 20 or more years of insertion experience versus those with less than 20, and this study compared third- and fourth-year surgeons to senior surgeons, which was suggested as a factor affecting success rates in the multivariate analysis. The results of this study, OR, 2.875; 95% CI, 1.202–8.534, may be influenced by the years of doctors’ experience. Therefore, further intensified investigations are warranted to elucidate the influence of the dominant hand on screw insertion.

## 5. Conclusions

In this study, we compared the success rates of left- and right-side miniscrew insertion by right-handed operators. The success rate for the right side was higher than that for the left side. The success rate was higher in the motorized group than in the manual group. In the manual group, the right side had a higher success rate than the left. In the motorized group, no difference was observed in the success rates between the left and right sides. For specific sites, the manual group had a lower success rate between the second premolar and the first molar on the left side of the maxillary arch. Multiple logistic regression analysis indicated that both the insertion side and the operator influenced the success rate. The use of manual screwdrivers leads to varying success rates between the left and right sides.

## Figures and Tables

**Table 1:** Demographic variables.

Variables	Number of Patients (Percentage)
Sex	
Male	53 (21.2)
Female	197 (78.8)
Mean age (years)	26.6
Age	
15–19	51 (20.4)
20–29	139 (55.6)
≥30	65 (26.0)

Values are shown in numbers (%).

**Table 2:** Values for the independent variables assigned in the multiple logistic regression analysis.

Independent Variables	Values
Age (>15 years old)	Primary value
Sex	0 = Male, 1 = Female
Insertion side	0 = left, 1 = right
Insertion site	0 = 6–7, 1 = 5–6
Operator’s years of experience	0 = 3–4 years, 1 = ≥5 years

Abbreviations: 5–6 between the second premolar and the first molar; 6–7 between the first molar and the second molar.

**Table 3:** Success rates by insertion method.

	Manual Driver	Motorized Driver	Total	Probability
Success	269 (77.7)	91 (84.2)	360 (79.2)	0.173
Failure	77 (22.2)	17 (15.7)	94 (20.7)	
Total	346 (100)	108 (100)		

Values are shown in numbers (%).

**Table 4:** Success rates by patient’s sex.

Method		Male	Female	Total	Probability
Manual driver	Success	59 (79.7)	210 (77.2)	269	0.75
	Failure	15 (20.3)	62 (22.8)	77	
	Total	74 (100)	272 (100)	346	
Motorized driver	Success	23 (82.1)	68 (85.0)	91	0.77
	Failure	5 (17.9)	12 (15.0)	17	
	Total	28 (100)	80 (100)	108	

Values are shown in numbers (%).

**Table 5:** Success rates by patient’s age.

Age (years)		Manual Driver	Motorized Driver	Total	Probability
<20	Success	60 (78.9)	12 (80.0)	72 (79.1)	0.99
	Failure	16 (21.1)	3 (20.0)	19 (20.9)	
20–29	Success	136 (77.2)	62 (83.7)	198 (79.2)	0.306
	Failure	40 (22.8)	12 (16.3)	52 (20.8)	
≥30	Success	73 (81.1)	17 (89.4)	90 (79.6)	0.353
	Failure	21 (18.9)	2 (10.6)	23 (20.4)	

Values are presented in number (%).

**Table 6:** Left–right differences in success rate by insertion method.

Insertion Method		Right Side	Left Side	Probability
Manual driver	Success	139 (82.7)	130 (73.0)	0.038 *
	Failure	29 (17.2)	48 (26.9)	
Motorized driver	Success	44 (84.6)	47(83.9)	0.99
	Failure	8 (15.3)	9 (16.0)	
Total	Success	185 (84.1)	177 (75.6)	0.049 *
	Failure	37 (16.8)	57 (24.3)	

Values are presented in numbers (%). *: *p* < 0.05.

**Table 7:** Left–right differences in success rates by insertion site.

Insertion Method	Site		Right Side	Left Side	Probability
Manual driver	5–6	Success	126 (82.3)	113 (71.9)	0.03 *
		Failure	27 (17.7)	44 (28.1)	
	6–7	Success	13 (86.6)	17 (80.9)	0.99
		Failure	2 (13.4)	4 (19.1)	
Motorized driver	5–6	Success	32 (84.2)	34 (82.9)	0.99
		Failure	6 (15.8)	7 (17.1)	
	6–7	Success	12 (85.7)	13 (86.6)	0.99
		Failure	2 (14.3)	2 (13.4)	

Values are presented in numbers (%). *: *p* < 0.05. Abbreviations: 5–6 between the second premolar and the first molar; 6–7 between the first molar and the second molar.

**Table 8:** Odds ratios of the independent variables for success according to multiple logistic regression analysis.

Independent Variables	Parameter Estimate	Odds Ratio	95% CI	Probability
Age (>15 years old)	−0.011	0.989	0.9632–1.013	0.39
Sex	0.120	1.128	0.6520–2.020	0.67
Insertion side	−0.506	0.602	0.3754–0.9573	0.03 *
Insertion site	0.459	1.584	0.7967–3.448	0.21
Operator’s years of experience	1.056	2.875	1.202–8.534	0.03 *

Filter was set with “success” as 0, while “failure” as 1. Abbreviation: CI, confidence interval. *: *p* < 0.05.

## Data Availability

All data generated and/or analyzed in the current study are included in this article.
